# Supplementary figures and images for: Comparative Proteomic Analysis of Transcriptional and Regulatory Proteins Abundances in S. lividans and S. coelicolor Suggests a Link between Various Stresses and Antibiotic Production
Source: Int J Mol Sci. 2022 Nov 26;23(23):14792. doi: 10.3390/ijms232314792 (PMC9739823; doi:10.3390/ijms232314792)

## Figure S1

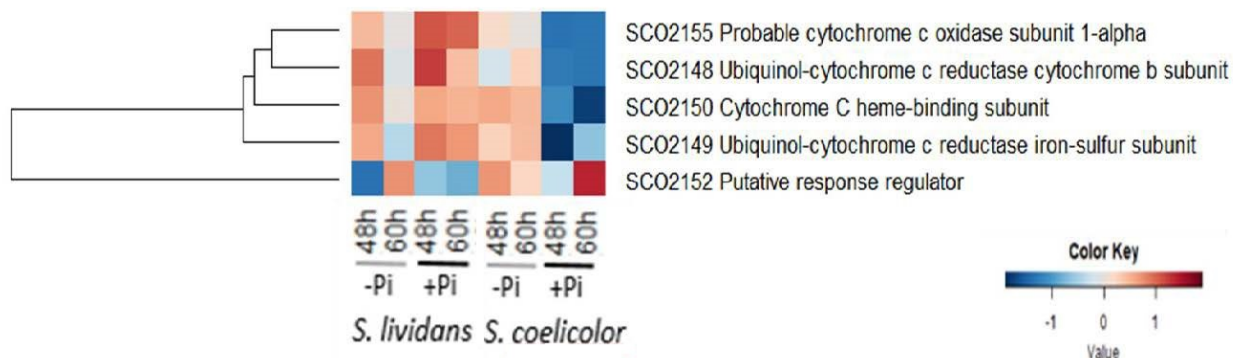

Supplement: Supplementary file 1 [file ijms-23-14792-s001.zip › ijms-2024041-Figure S1.pdf]
